# Preparation and In Vitro-In Vivo Evaluation of Luteolin Loaded Gastroretentive Microsponge for the Eradication of *Helicobacter pylori* Infections

**DOI:** 10.3390/pharmaceutics13122094

**Published:** 2021-12-06

**Authors:** Mohammed Jafar, Mohammed Salahuddin, Mohd Sajjad Ahmad Khan, Yasir Alshehry, Nazar Radwan Alrwaili, Yazeed Ali Alzahrani, Syed Sarim Imam, Sultan Alshehri

**Affiliations:** 1 Department of Pharmaceutics, College of Clinical Pharmacy, Imam Abdulrahman Bin Faisal University, P.O. Box 1982, Dammam 34212, Saudi Arabia; yaalshehry@iau.edu.sa (Y.A.); 2170000180@iau.edu.sa (N.R.A.); 2170006136@iau.edu.sa (Y.A.A.); 2 Department of Clinical Pharmacy Research, Institute for Research and Medical Consultations (IRMC), Imam Abdulrahman Bin Faisal University, P.O. Box 1982, Dammam 34212, Saudi Arabia; msalahuddin@iau.edu.sa; 3 Department of Basic Sciences, Deanship of Preparatory Year and Supporting Studies, Imam Abdulrahman Bin Faisal University, P.O. Box 1982, Dammam 34212, Saudi Arabia; mskhan@iau.edu.sa; 4 Department of Pharmaceutics, College of Pharmacy, King Saud University, P.O. Box 2457, Riyadh 11451, Saudi Arabia; simam@ksu.edu.sa (S.S.I.); salshehri1@ksu.edu.sa (S.A.)

**Keywords:** luteolin, buoyancy, *H. pylori*, microparticulate system, in vivo study

## Abstract

The current study aimed to develop a luteolin gastric floating microsponge for targeting *Helicobacter pylori*. The microsponge formulations were prepared by a quasi-emulsion method, and then evaluated for various physicochemical variables. The best microsponge was further assessed for drug-polymer interactions, surface morphology, in vivo floating, and in vitro anti *H. pylori* activity. The formulation which exhibited comparatively good production yield (64.45% ± 0.83), high entrapment efficiency (67.33% ± 3.79), prolonged in vitro floating time (>8 h), and sustained in-vitro drug release was selected as the best microsponge. The SEM study revealed that the best microsponge was spherical in shape and has a porous surface with interconnecting channels. DSC and XRD studies demonstrated the dispersion of luteolin in the polymeric matrix of the microsponge. Ultrasonography confirmed that the best microsponge could in the rat stomach for 4 h. The in vitro MIC results indicate that the anti *H. pylori* activity of the best microsponge was almost doubled and more sustained compared to pure luteolin. To conclude, it can be said that the developed luteolin gastric floating microsponge could be a better option to effectively eradicate *H. pylori* infections and the histopathological and pharmacodynamic assessments of our best microsponge can be expected to provide a rewarding outcome.

## 1. Introduction

*Helicobacter pylori* (*H. pylori*) is a bacterium that colonizes the human stomach. Around 70% of the world’s population are infected with *H. pylori* and are asymptomatic [1]. *H. pylori* infection is the main cause of gastric ulcers and gastritis that can lead to gastric cancer [2,3,4]. Even though more than half of the human population is infected with *H. pylori* only 10–20% will develop a peptic ulcer, and only 1–2% are at risk to develop gastric cancer or MALT lymphoma. The antibiotics which are currently in use in the treatment of *H. pylori* infections are amoxicillin, tetracycline, metronidazole and macrolides (azithromycin or clarithromycin). Despite the successful use of these treatments, 20% of patients fail to respond positively to therapy. Therapy failure is mostly due to the reduced efficacy of the above antibiotics. Moreover, *H. pylori* develop resistance by point mutations and thus shows resistance to metronidazole and clarithromycin in particular, which has high clinical significance, as both drugs are considered to be the first-choice antibacterial treatment for the eradication of *H. pylori*. Thus, the resistance to these antibiotics has led to the use of other medications, for instance, amoxicillin-levofloxacin and amoxicillin-rifabutin. Even though these medications can effectively eradicate some resistant *H. pylori*, there are some limitations on medication availability and the high cost of these medications in developing countries. Therefore, nonantibiotic therapies such as phytotherapy and probiotics are considered the best choice for the treatment of *H. pylori* [1].

Probiotics reduce side effects and enhance the efficacy of antibiotics, perhaps because they mimic the function of the human microbiota. Phytotherapy is the use of plant extracts and chief phytoconstituents as medicines [1]. Phytotherapy is an interesting and promising approach as plants contain biochemicals that are considered safer to use in human beings and commonly act at different and novel target sites unlike synthetic antibiotics, therefore they decrease the possibility for the development of resistance to *H. pylori* [4]. Low cost, high efficacy, good bioavailability and better tolerability have led researchers to focus more on this area [5]. Flavonoids are a large group of plant chemicals that are found in many vegetables and fruits. In previous studies, flavonoids showed the ability to decrease the risk of chronic diseases, including *H. pylori* and other severe microbial infections. Flavonoids are commonly used plant constituents in traditional medicine to treat and prevent several diseases. Luteolin (LUT) is one of the commonest flavonoids found in edible plants and it has numerous biological activities that include anti-inflammatory, antioxidant, anti-cancer, and anti-microbial properties [6]. In a recent study, luteolin was tested on different strains of *H. pylori* resistant to erythromycin, metronidazole, levofloxacin, and azithromycin. The study results were promising as luteolin showed growth inhibitory effect towards both antibiotic-susceptible and antibiotic-resistant strains of *H. pylori*. Additionally, luteolin has bactericidal activity against antibiotic-resistant strains of *H. pylori* [4].

Oral drug delivery systems are superior and the most common drug delivery systems for drug administration due to the ease of administration, storage, transport, cost-effectiveness, and high patient compliance. However, the oral drug delivery system has issues such as low bioavailability, small surface area, high enzymatic activity, and short gastric retention time. Conventional drug delivery systems cannot overcome the incomplete release of drugs, decrease in drug efficacy, and frequent dosing of drugs due to the problems of the GIT stated above. Therefore, the development of gastroretentive drug delivery systems has accomplished several advantages over the conventional dosage forms such as prolonged gastric residence time of the dosage form, improving the drug absorption, and the ability for targeted and local delivery of drugs in the stomach [7]. To our knowledge, no studies focusing on treating the *H. pylori* infection using gastroretentive microparticulate systems are reported in the existing literature.

The microsponge is one of the modern matrices gastroretentive microparticulate systems having a spherical structure and composed of interconnected channels that form a rigid-long lasting structure with a large porous surface [8]. These microparticulate structures are 5–300 µm in diameter and have a pores size of less than 0.25 µm. Microsponge systems are available in different dosage forms for systemic (e.g., tablets and capsules) and topical (gel and emulsion) action [9]. Microsponge systems have demonstrated superior advantages over other gastroretentive systems including minimal dose dumping, high entrapment efficiency 50–60%, increased drug thermal, physical, and chemical stability, controlled and sustained release, free-flowing properties, self-sterilization, and compatibility with various ingredients and vehicles. This system does not only offer pharmaceutical advantages but also an economic advantage. The microsponge system is considered cost-effective as compared to other systems, including nanocapsules and liposomes [9,10,11].

**Figure 1:**
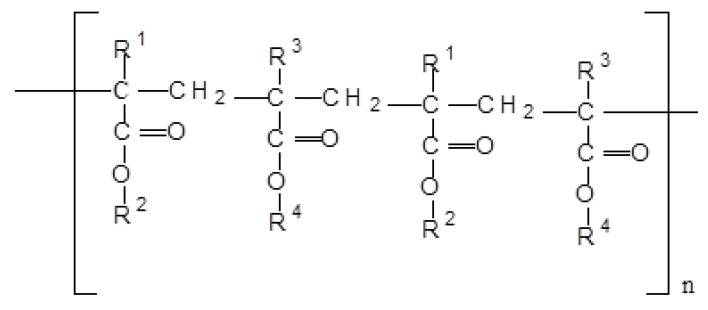
Chemical structure of Eudragit RS 100. Note: R^1^ = H, CH_3_ R^2^ = CH_3_, C_2_H_5_ R^3^ = CH_3_ R^4^ = CH_2_CH_2_N(CH_3_)_3_^+^Cl^−^.

Eudragits are synthetic polymers obtained by polymerization of acrylic acid and methacrylic acids or their esters like butyl ester or dimethyl aminoethyl ester. Eudragit RS (EGT) polymers are polycations consisting of poly (ethyl acrylate), methyl methacrylate, and trimethylammonium-ethyl methacrylate chloride with the ratio of 1:2:0.2 and 1:2:0.1, respectively (Figure 1). Increasing the amount of quaternary ammonium groups renders the polymer network more hydrophilic. Eudragit RS polymers contain only 5% hydrophilic quaternary ammonium groups, far less than any other eudragit, so they are insoluble in acidic digestive fluids but are permeable and demonstrate pH-independent swelling and matrix structure forming ability which is considered as an ideal characteristic of this polymer to be utilized in the design of prolonged release matrix type of drug delivery systems [12,13].

**Figure 2:**
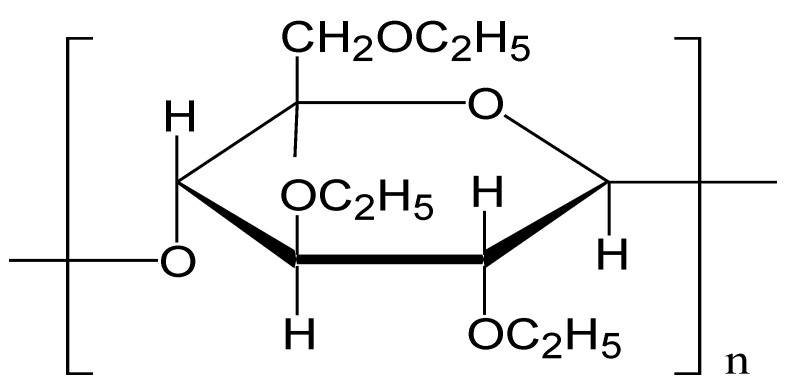
Chemical structure of ethyl cellulose.

Ethyl cellulose (EC), an ethyl ether of cellulose, is a hydrophobic, free-flowing, white powder prepared from wood pulp or cotton. Pharmacopeial monographs describe EC as partially *O*-ethylated cellulose (Figure 2). The use of EC in the formulation development of different pharmaceutical delivery systems, mainly in oral dosage forms, is not a new approach for several pharmaceutical industries across the globe. Its hydrophobic nature and swelling ability in the gastrointestinal fluid make it a potential candidate to modify and enhance the biological performance of drug delivery systems. The primary focus of EC utilization is the design of drug delivery systems with sustained drug release (SR), as EC ensures the complete dissolution and release of drug throughout the gastrointestinal tract, thus maintaining the concentration of drug constant and improving the patient compliance by reducing the frequency of drug administration, therefore enhancing the effectiveness of the pharmacotherapy [14].

Both eudragit and ethyl cellulose possess some common characteristics such as low density, low tissue toxicity, and high biological stability mainly in the acidic environment so they have been successfully utilized alone or in combination in the design of several gastric floating systems, including microspheres [15]. Therefore, our research aims to develop a gastric floating microsponge formulation of luteolin utilizing Eudragit RS 100, and ethyl cellulose polymers for targeting and local effect in the stomach for the effective eradication of *H. pylori* infections.

## 2. Material and Methods

### 2.1. Material

Luteolin and Eudragit RS100 were procured from Beijing Mesochem Technology. Beijing, China and UFC Biotechnology, Amherst, NY, USA. Ethyl cellulose, tween-80, acetone and hydrochloric acid were purchased from SD Fine Chemicals Pvt. Ltd. Mumbai, India. A reference strain (*H. pylori* ATCC43504) from the American Type Culture Collection (Manassas, VA, USA) was used in the study. Brain heart infusion broth/agar media and bovine serum albumin were obtained from Sigma (St. Louis, MO, USA). Wistar albino rats were used for the in vivo study. All other chemicals used were of analytical research grade.

### 2.2. Preparation of Luteolin Gastric Floating Microsponge

Luteolin (LUT) is a water-insoluble drug [16], so a quasi-emulsion method [8] was used to prepare its gastric floating microsponge. Preparation of LUT microsponge involves three steps. In the first step an organic phase was prepared in a glass beaker by dissolving in a fixed volume of acetone, fixed amounts of LUT and varying amounts of the polymers Eudragit RS 100 (EGT) and ethyl cellulose (EC) (Table 1). In the second step, an aqueous phase was prepared in another glass beaker by dissolving an emulsifying agent (0.6% *w*/*v* Tween-80) in distilled water. In the final step, the organic phase was transferred dropwise to the beaker containing an aqueous phase with continuous stirring at 1500 rpm in an overhead stirrer (Eurostar 20, IKA, Staufen, Germany) for 90 min. Finally, the LUT gastric floating microsponges suspended in the aqueous phase were then filtered, air-dried and preserved in a refrigerator until used for further studies.

### 2.3. Physicochemical Evaluation of LUT Gastric Floating Microsponge

#### 2.3.1. Production Yield

To estimate the preparation method efficiency, individual quantities of LUT, EGT, and EC of all microsponge formulations were weighed individually on a calibrated digital weighing balance and their weights were noted as theoretical weight. Next, the prepared corresponding LUT gastric floating microsponge formulations were weighed and their weights were noted as practical weight. The following formula was used for production yield calculations:

$$\mathrm{Production}\;\mathrm{yeild}=\frac{\mathrm{Practical}\;\mathrm{weight}\;\mathrm{of}\;\mathrm{the}\;\mathrm{final}\;\mathrm{microsponge}}{\mathrm{Theoretical}\;\mathrm{weight}\;\left(\mathrm{LUT}+\mathrm{EGT}+\mathrm{EC}\right)}\times 100$$

#### 2.3.2. Drug Content and Entrapment Efficiency

Weighed amounts of each LUT gastric floating microsponge formulation equivalent to 10 mg of LUT, were dissolved in 100 mL of pH 1.2 media in separate 100 mL volumetric flasks and then all the flasks were sonicated for 12 h in a water bath. Next, using Whatman filter paper, the media were filtered and subsequently diluted with pH 1.2 solution to prepare 20 mcg/mL concentration sample solutions and then the absorbance of each sample solution was recorded at 347 nm using a double beam UV-Visible spectrophotometer (Shimadzu 1700, Kyoto, Japan). The following equations were used to calculate % drug content and % entrapment efficiency of each LUT gastric floating microsponge formulation:

$$\%\;\mathrm{Drug}\;\mathrm{content}\;=\frac{\mathrm{Actual}\;\mathrm{amount}\;\mathrm{of}\;\mathrm{LUT}\;\mathrm{in}\;\mathrm{microsponge}}{\mathrm{Weighed}\;\mathrm{amount}\;\mathrm{of}\;\mathrm{microsponge}}\times 100$$

$$\%\;\mathrm{Entrapment}\;\mathrm{efficiency}\;=\frac{\mathrm{Actual}\;\mathrm{amount}\;\mathrm{of}\;\mathrm{LUT}\;\mathrm{in}\;\mathrm{microsponge}}{\mathrm{Theoretical}\;\mathrm{amount}\;\mathrm{of}\;\mathrm{LUT}\;\mathrm{in}\;\mathrm{microponge}}\times 100$$

#### 2.3.3. In Vitro Floating Study

Floatability of LUT gastric floating microsponge formulations was assessed as per the reported method [17]. A USP type II dissolution test apparatus (USP XXIV8 basket Dissolution Test Station, Electrolab Pvt. Ltd., Mumbai, India) was used to assess the floatability of the LUT gastric floating microsponge formulations. The dissolution flasks of the apparatus were filled with 900 mL dissolution media (pH 1.2), maintained at a temperature of 37 ± 0.5 °C, rotated at 50 rpm and amounts of the LUT gastric floating microsponge formulations equivalent to 10 mg LUT were then introduced in the dissolution flasks. Floating lag time, being the time microsponges took to float on the surface of the media, and the total duration of floating (floating log time) were measured using a stopwatch.

#### 2.3.4. Size Determination

The dynamic light scattering method coupled with a computerized detection and inspection system (Malvern Zetasizer, Malvern, UK) was used to determine the size of the prepared microsponge formulations.

### 2.4. In Vitro Drug Release Study

The in-vitro drug release studies of pure LUT, and LUT gastric floating microsponge formulations were carried out using a USP type II automated dissolution test apparatus (LOGAN Instruments Corp., Somerset, NJ, USA). A pre-soaked cellophane membrane (Himedia Pvt Ltd., Mumbai, India) was used to wrap the LUT gastric floating microsponges and the membrane was tied to the paddle and the paddle was immersed in 900 mL of the release media (pH 1.2). The temperature of the media was maintained at 37 ± 0.5 °C and the paddles were rotated at a low speed of 50 rpm to minimize the chances of occurrence of damage to the microsponge structure due to its prolonged exposure to stirring [18]. The samples were withdrawn automatically through the autosampler at preset time intervals for 24 h to evaluate the cumulative amount of LUT released from the pure LUT and its microsponge formulations by recording their absorbances using a double-beam UV–visible spectrophotometer (Shimadzu 1700) at 347 nm. A fresh medium of volume equivalent to that of the volume of the samples withdrawn was transferred automatically by the dissolution test apparatus to the flasks to maintain the sink condition. The recorded absorbances of the samples were used to calculate the cumulative amount of drug released from each sample as well as the mechanism of LUT released, using PCP Disso V3 Software (Program developed by Bharati Vidyapeeth Deemed University, Pune College of Pharmacy, India).

### 2.5. Scanning Electron Microscopy (SEM)

In this study, a scanning electron microscope (JSM 6360A, JEOL, Tokyo, Japan) was used to capture the images of LUT, EGT, EC, LUT/EGT/EC gastric floating microsponge formulation (F-3). With the help of a double-sided tape, the sample was kept on the brass stub and was coated with a thin layer of gold by ion sputter and then it was scanned under an electron microscope at different magnification power, at an electric voltage of 20 kV and the images of each sample were captured and saved in the system. These images were used to study the surface morphology of the LUT gastric floating microsponge formulation.

### 2.6. Differential Scanning Calorimetry (DSC)

In this study, a differential scanning calorimeter (DSC 214 Polyma Netzsch, Selb, Germany) was used to record the thermograms of LUT, EGT, EC, LUT/EGT gastric floating microsponge formulation (F-1), and LUT/EGT/EC gastric floating microsponge formulation (F-3). 4 to 5 mg of the sample was weighed in DSC aluminium pan and then the pan was hermetically sealed. The sealed pans were then transferred to the DSC instrument for the recording of thermograms of the samples. The DSC measurements of each sample were performed in a nitrogen atmosphere (40 mL/60 mL min^−1^) between 0 and 400 °C at a heating rate of 10 °C min^−1^.

### 2.7. X-ray Diffractometry (XRD)

An X-ray diffractometer (Ultima IV, Rigaku Inc., Tokyo, Japan) was used to record the XRD pattern of LUT, EGT, EC, LUT/EGT gastric floating microsponge formulation (F-1), and LUT/EGT/EC gastric floating microsponge formulation (F-3). In the XRD instrument, CuKa radiation was used in the wavelength 1.54060 A0 to measure the samples. Each sample was step scanned between 0 and 700 at 2θ scale while measuring the intensities of the diffraction peaks.

### 2.8. In Vivo Floating Study in Albino Rats

After the approval of the institutional review board of Imam Abdulrahman Bin Faisal University, Dammam, Saudi Arabia, under the approval number IRB-UGS-2021-05-135 and procuring the animals from the animal house of the Institute of Research and Medical Consultation Studies of the above university, this study was conducted on three Wistar albino rats. The rats, weighing 180–200 g, were housed individually in metabolic cages and maintained at 25–30 °C, 12 h light and 12 h dark cycle. The rats were fasted for 18 h before the administration of the LUT gastric floating microsponge formulation (F-3). The first control animal had only access to water during the experiment while the second and the third animals were orally administered the selected LUT gastric floating microsponge formulation (F-3) equivalent to 10 mg/kg of LUT. Before ultrasound scanning the rats were anaesthetized, and their thick hairs were removed using electric hair clippers and finally a depilatory cream was applied to the scanning area. The purpose of applying cream on the rat’s skin is to prevent trapping air bubbles under any remaining hair stubble [19]. After applying the cream, the stomach region’s ultrasound images were taken using an ultrasound machine (esaote veterinary, MyLabOneVET, Genova, Italy) to monitor the floating behavior of the administered LUT gastric floating microsponge formulation (F-3). Image of the first rat was taken on an empty stomach and noted as 0 h, whereas images of second and third rats were taken at 2 h and 4 h after the administration of the LUT gastric floating microsponge formulation.

### 2.9. In Vitro Anti H. pylori Activity

#### 2.9.1. Determination of Minimum Inhibitory Concentration (MIC)

The MICs of LUT and the selected LUT gastric floating microsponge formulation (F-3) were evaluated against the ATCC43504 *H. pylori* strain using a microdilution broth assay. Stock solutions of the samples were prepared in 1% DMSO. The test strain was grown on brain heart infusion broth supplemented with 7% bovine serum albumin and incubated under microaerophilic conditions for 3 days. Two-fold serial dilutions of the test samples were prepared in 96 well microtiter plates. The dilutions were prepared in phosphate buffer saline, 100 µL of Mueller-Hinton broth containing test samples ranging from 0.06 µg/mL to 16 µg/mL. 0.1 µL of *H. pylori* suspension (107 cfu/mL) was added and then incubated in a microaerophilic environment (CO_2_ 10%, O_2_ 5%, N_2_ 85%) at 37 °C for 5 days. MICs of samples were considered as the lowest concentration at which the visible turbidly of bacterial growth was inhibited.

#### 2.9.2. Determination of Duration of Growth Inhibition

The test strain (107 cfu/mL) was treated with 2× MIC of test samples and incubated in 200 µL of Mueller-Hinton broth at 37 °C for 96 h under a microaerophilic environment (CO_2_ 10%, O_2_ 5%, N_2_ 85%). The duration of action of samples was recorded as the period after which visible turbidity appears for bacterial growth.

### 2.10. Statistical Analysis

The experimental data obtained in this study was evaluated using the one-way analysis of variance test (ANOVA). Further, Student’s *t*-test was conducted for comparisons among the standard and the microsponge formulation test groups and the significance levels were reflected at *p* < 0.05.

## 3. Results and Discussion

### 3.1. Physicochemical Evaluation of LUT Gastric Floating Microsponge

Various experiments demonstrated the variable product yield (PY), drug content (DC), and entrapment efficiency (EE) for luteolin (LUT) gastric floating microsponge formulations. However, all microsponge formulations floated within no time (lag time is zero seconds) after placing them in the medium and they remained floating in the medium for more than 8 h (log time). The PY, DC, and EE values were found in the range of 30.94% ± 0.33 to 97.59% ± 0.54, 24.67% ± 1.76 to 33.67% ± 1.89 and 49.33% ± 3.51 to 67.33% ± 3.79 respectively for the microsponge formulations (Table 2). Agglomeration and sticking of the polymers on the stirrer blades and inside walls of the beaker and also to the glass rods could be the reason for the low production yield of the microsponge formulation. The product yield was also found to be dependent on the choice of the polymer. The yield of the microsponge containing EGT alone (F-1) was the highest, whereas, the yield of the microsponge formulations containing both EGT and EC was decreased by decreasing the concentration of EGT and increasing the concentration of EC in the formulations (F-2 to F-4). The lowest yield was recorded for the microsponge containing EC alone (F-5). This may be due to the movement of EC into continuous phase and thus forming thick agglomerates accompanied with sticking of the polymer to the stirrer blade and beaker surface and glass rods. These findings of the LUT microsponge yield are like the ones that appeared in the past reports on the related research [16,20].

It has been observed that the DC and EE of the microsponge formulations containing both EGT and EC polymers have been increased with an increase in the concentration of EC in the formulations (Table 2). The DC and EE values obtained for the microsponge formulation (F-3) containing equal concentrations of both polymers were found to be 33.67% ± 1.89 and 67.33% ± 3.79, respectively, and these readings are highest compared to other formulations containing both polymers. This may be attributed to the highest combined effects of matrix formation and swelling by the EGT and EC respectively. It has been reported that when two hydrophobic polymers are used in combination with proper proportion in the microparticulate systems they could greatly influence the drug entrapment efficiency of that system [21]. The entrapment efficiency results of our F-3 formulation are in compliance with the study reporting on the combined effects of EGT and EC on microparticulate systems of a drug having almost similar physical nature to LUT [22]. The EE of the microsponge containing EGT alone (F-1) was found to be the lowest (49.33% ± 3.51) as EGT was unable to form a gel-like structure, unlike EC. The DC results obtained for the formulations containing EGT alone and EC alone are in accordance with the reported findings [23].

As far as the particle size of the microsponge formulations is concerned it will vary by varying the stirring speed, amounts of the polymers, and emulsifying agent in the formulations [8,23]. In our case there was no significant difference observed in the size of the prepared microsponge formulations and it was in the range of 3.36 µm to 3.61 µm size (Table 2). The reason attributed could be the same stirring speed used during the preparation of all the microsponges. Furthermore, 1:1 drug to polymer ratio i.e., the presence of the same amounts of either single polymer or the combination of two polymers of almost similar nature and also addition of the same amounts of emulsifying agent in all formulations (Table 1) could be the other contributing factors for the sizes obtained for the microsponges. These results are in accordance with previously reported results [24]. Most of the reported microsponges demonstrated sizes between 5 µm to 300 µm and one of the reasons stated for these sizes could be the use of higher amounts of swellable polymer(s) which are forming highly viscous droplets during the microsponge preparation resulting in the formation of large size microsponges [8,10,23].

### 3.2. In Vitro Drug Release Study

**Figure 3:**
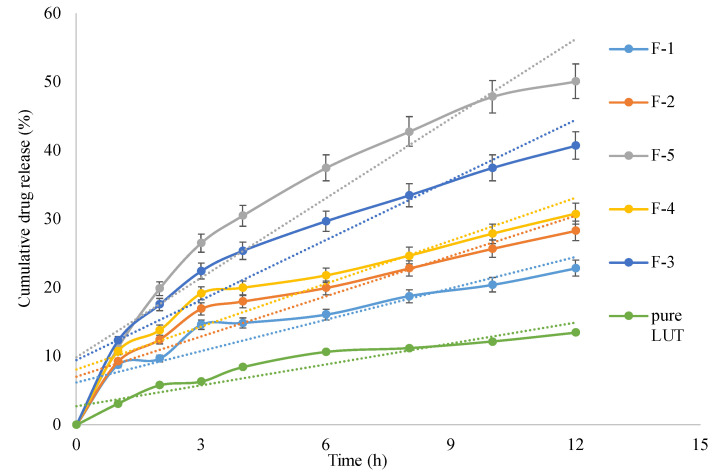
Cumulative drug release Vs time profile of pure LUT, and LUT gastric floating microsponge formulations (F-1 to F-5). Each study performed in triplicate and data shown as mean ± SD (n = 3).

The in vitro drug release obtained for the pure LUT was just 13.46% ± 0.79 in 12 h. This low drug release could be due to its poor aqueous solubility [16]. The highest in vitro cumulative LUT release (50.22% ± 1.07) from F-5 and the lowest release (20.42% ± 0.98) from F-1 was obtained at the end of 12 h (Figure 3). However, the microsponge formulations containing both EGT and EC polymers (F-2 to F-4) showed increased drug release rates with an increase in the amount of EC and decrease in the amount of EGT, but their release rates were less than F-5 formulation containing EC alone and more than F-1 formulation containing EGT alone. This pattern of drug release from these different microsponge formulations is attributed to the fact that EGT, an anionic copolymer of methacrylic acid and methyl methacrylate containing free carboxylic and ester groups, is insoluble in acidic medium and also exhibits low permeability [25], whereas EC, despite its low solubility at all pH environments, swells and forms highly porous gel structure in gastric acidic pH medium [15] and therefore it showed higher rates of drug release than EGT. Furthermore, it is evident from the literature that combining EGT and EC in a proper proportion in a sustained release drug delivery system could significantly influence the release of drug from the newly formed polymeric matrix structure and this drug release is not necessarily the sum of the drug release exhibited by the same systems containing same individual polymers [26]. Nevertheless, all microsponge formulations exhibited higher rates and extent of drug release compared to pure LUT indicating that the aqueous solubility of LUT was significantly enhanced due to its micronization and molecular dispersion in the polymeric network and nanopores formed by the microsponge [8]. The drug release mechanism from the microsponge could be understood by fitting the in vitro release models on the in vitro drug release profiles. The release of LUT from all microsponges could be best described by Higuchi’s diffusion kinetics model. The diffusion exponent (n) value obtained for all microsponge formulations was found to be less than 0.5. This value clearly tells us that the LUT release mechanism from all microsponge formulations is Fickian diffusion and it is controlled by the porous structure of the microsponge preparations.

### 3.3. Scanning Electron Microscopy (SEM)

**Figure 4:**
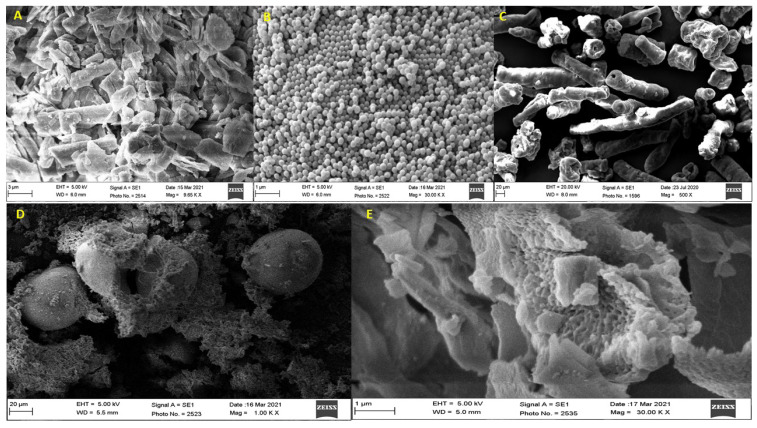
SEM images of (**A**) Luteolin (LUT), (**B**) Eudragit RS100 (EGT), (**C**) Ethyl cellulose (EC) (**D**) LUT microsponge (Shape view), (**E**) Ruptured microsponge (Surface view).

The micrographs of LUT (Figure 4A) revealed the crystalline nature of the drug. Figure 4B,C reveal the shape and surface morphology of the polymers EGT and EC, respectively. Figure 4D reveals that the microsponge was spherical in shape. High-resolution micrographs of the microsponge formulation (Figure 4E) showed the ruptured microsponge revealing the porous polymeric matrix of the microsponge and affirming its internal structure. Moreover, few particles of LUT adhering to the internal surface of the microsponge can also be seen in the same micrograph, which indicates that most of the LUT was amorphized and molecularly dispersed in the porous polymeric matrix of the microsponge and only a small amount in the semi-crystalline state has adhered to the internal surface of the microsponge.

### 3.4. Differential Scanning Calorimetry (DSC)

**Figure 5:**
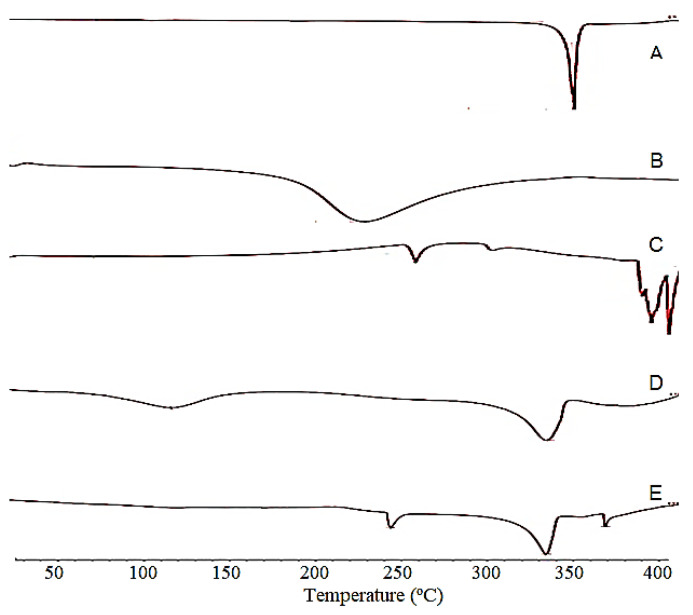
DSC thermograms of (**A**) Luteolin (LUT), (**B**) Eudragit RS100 (EGT), (**C**) Ethyl cellulose (EC) (**D**) LUT microsponge (F-1), (**E**) LUT microsponge (F-3).

LUT, EGT, EC, and the LUT microsponge (Formulation-3) samples were tested by DSC. As depicted in Figure 5, the DSC thermogram of LUT and EGT showed endothermic peaks at 341.0 °C, and 130.9 °C, respectively, which correspond to their melting points and indicate their crystalline and amorphous natures, respectively. EC showed no endothermic peaks indicating it is in an amorphous form. In each of the DSC thermograms of the microsponge formulations, the characteristic endothermic peak of the LUT was observed, thus indicating that there was compatibility between the drug and the polymers used. A significant decrease in the melting point of the LUT in the microsponge formulations was observed, that could be due to the reduction in the crystallinity of the drug due to entrapment in the porous polymeric matrix of the microsponge.

### 3.5. X-ray Diffractometry (XRD)

**Figure 6:**
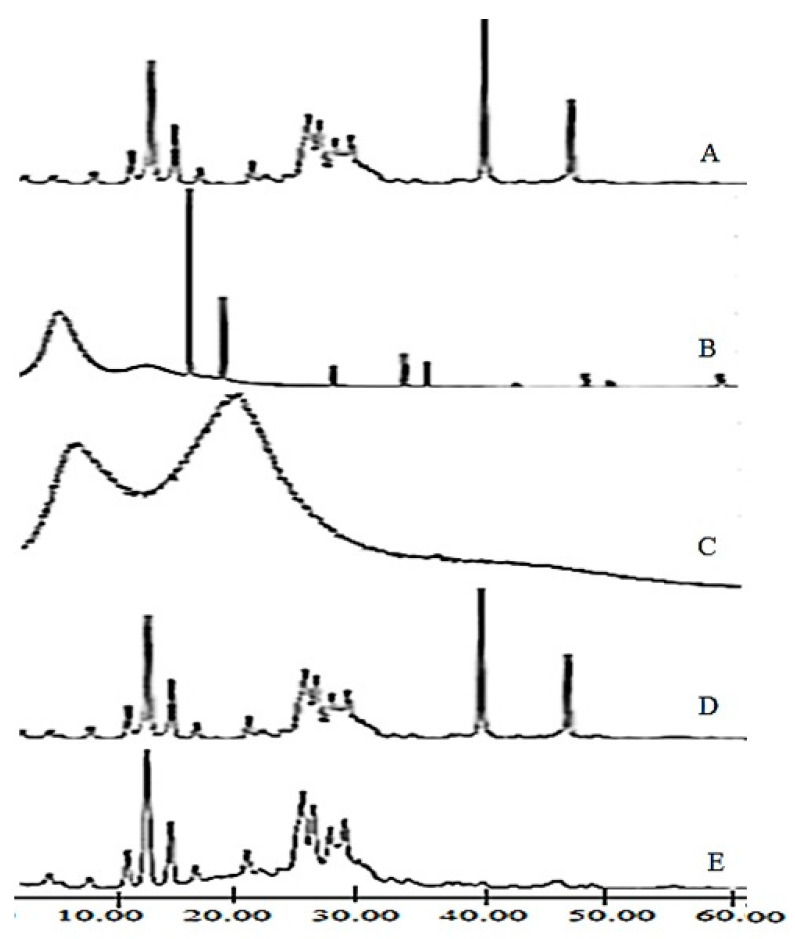
X-ray diffractograms of (**A**) Luteolin (LUT), (**B**) Eudragit RS100 (EGT), (**C**) Ethyl cellulose (EC) (**D**) LUT microsponge (F-1), (**E**) LUT microsponge (F3).

The XRD spectra of LUT showed various intense peaks at 14.3°, 16.1°, 26.1°, 29.2°, and 27.6°, suggesting that LUT is present in a crystalline form [27]. Both EGT and EC showed no characteristic peaks at a diffraction angle range of 5–40° indicating that they are amorphous [28,29]. The X-ray diffractograms of LUT microsponge formulations were characterized by a significant decrease in the intensity of the distinct diffraction peaks of LUT, indicating a drug semi amorphization or its partial dissolution in the amorphous polymer(s) (Figure 6). F-3 was found to be more amorphous compared to F-1 because some sharp peaks observed in F-1 were absent in F-3. These XRD results are consistent with the DSC study findings.

### 3.6. In Vivo Floating Study

**Figure 7:**
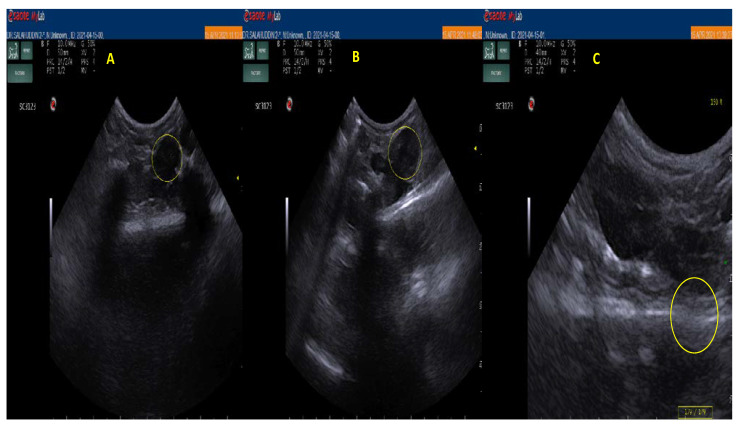
Ultrasonography images of rat stomach after administration of (**A**) clear liquid (**B**,**C**) LUT gastric floating microsponge (2nd and 4th hours respectively). Note: The yellow circle in the images shows the presence of microsponge in the upper GIT area of rats.

The main problem with the use of ultrasonography is that it has a comparatively low spatial resolution, but its high temporal resolution along with the latest technological advancements over the past years provide a basis for potential use for localization of gastroretentive drug delivery systems. New ultrasonographic systems are portable, wireless, and easy to operate. The intragastric imaging of drug delivery systems is likely restricted to slow or non-disintegrating systems [30]. As shown in Figure 7, the first ultrasonographic image of the rat stomach administered with clear liquid shows a stary night appearance indicating the presence of gas bubbles in the stomach, and the second and third images show lumps and coagulated mass of administered LUT microsponge formulation (F-3) at the 2nd and 4th hours, respectively. The main reason for running this experiment for such a short period of time i.e., until 4th hour is due to the fact that the gastric transit time is shorter (1 to 2 h) in rats. Thus, it is evident from the images that the formulation F-3 was retained in the rat’s stomach beyond the gastric transit time, and also, we could say that it was floating based on the fact that floating systems remain buoyant on gastric fluids and are less likely to be expelled from the stomach compared to other systems which lie in the distal part of the stomach and are propelled by the peristaltic movement. The in vivo floating results obtained for F-3 formulation are in agreement with the previous study reporting in-vivo floating of microsponges for 8 h after X-ray examination of the rats’ stomachs [31].

### 3.7. In Vitro H. pylori Activity

#### Determination of MIC and Duration of Action

*H. pylori* infections are leading causes for gastritis and peptic ulcer diseases. To combat these diseases, the available conventionally used antibiotics are amoxycillin, clarithromycin, tetracycline and metronidazole, but no therapy has proven to provide 100% *H. pylori* eradication rate because of low stability of these antibiotics at the low pH of gastric juice [32,33]. Metronidazole is found to be marginally affected by low pH and therefore, this drug is a preferred choice against *H. pylori* [33,34], but resistance against this drug in *H. pylori* has been reported [35]. In a study conducted by Byoungrak et al. [36], the MIC ranges for clarithromycin, amoxicillin, metronidazole, tetracycline, and levofloxacin were 0.016–0.125, 0.016–0.125, 64–256, 0.125–1 and 0.064–0.5 µg/mL against *H. pylori* ATCC 43504, respectively.

In this regard, in our study we also used *H. pylori* ATCC43504, a known metronidazole-resistant reference strain. This strain is however susceptible to other drugs like amoxicillin and clarithromycin [37]. As shown in Table 3, the MIC of luteolin (a flavone class phytochemical) against *H. pylori* ATCC43504 is found to be 2 µg/mL. Other studies have shown MICs for luteolin and other flavones like apigenin and chrysin against the drug resistant or susceptible *H. pylori* strains in the range of 4–32 µg/mL [37,38,39], whereas luteolin-microsponge exhibited a MIC of 4 µg/mL against the test strain. This indicates that, luteolin alone and luteolin-microsponge formulation are potential growth inhibitors against *H. pylori* strains at lower concentrations compared to other flavones and metronidazole as revealed from the mentioned studies. However, the duration of action of luteolin-microsponge formulation (up to 48 h) was observed to be higher than luteolin (24 h). In the present work, the optimized microsponge formulation of luteolin has exhibited in vitro antimicrobial activity comparable to the activity of standard luteolin against *H. pylori* strain. The two-fold increase in MIC of microsponge formulation of luteolin compared to pure luteolin could be attributed to the presence of other compounds viz. ethyl cellulose and eudragit in developed formulations. These compounds are biologically inert polymers that reduces the amount of active compound in the developed microsponges, i.e., luteolin, by half, as mentioned in Table 1. Therefore, the MIC of the developed microsponge is increased by two-fold. However, the test strain is found to be susceptible to luteolin in the developed formulation at a considerably lower concentration for a prolonged period.

We observed that in our study that the MICs of luteolin and its microsponge against metronidazole resistant *H. pylori* ATCC43504 are significantly low compared to the studies conducted for metronidazole against *H. pylori* strains [36,37]. Therefore, our study suggests that luteolin and a luteolin-based microsponge can be effectively used against drug-resistant or susceptible strains of *H. pylori*. Furthermore, the present study has highlighted that luteolin microsponge possesses potent and sustained release antibacterial activity against *H. pylori*. Therefore, it could be considered as a promising future alternative drug with increased bioavailability to be effectively exploited against the treatment of *H. pylori-induced* peptic ulcers.

## 4. Conclusions

A luteolin gastro-retentive microsponge with high production yield, acceptable drug content, high drug entrapment efficiency, sustained drug release, and prolonged in vivo floatability in the albino rat model was successfully developed. The emergence of antibiotic resistance in *H. pylori* has reduced the efficacy of conventional drugs such clarithromycin, amoxicillin, metronidazole, tetracycline and levofloxacin [36]. Additionally, antimicrobial studies conducted against a metronidazole-resistant *H. pylori* strain have also confirmed the potential and prolonged antimicrobial activity of the best luteolin microsponge formulation compared to luteolin alone. Thus, it could be concluded that the LUT gastro-retentive microsponge formulation appears to have promising potential for the targeted delivery of luteolin in the stomach region and also the effective eradication of *H. pylori* infections. However, further in vivo experiments are needed to evaluate in vivo the efficacy of our developed luteolin-microsponge formulation against *H. pylori* infections. Additional pharmacokinetics, histopathological and clinical assessments of our best microsponge are needed to recapitulate the many productive results.

## Figures and Tables

**Table 1:** Composition of LUT gastric floating microsponge.

Ingredients *	Formulations				
	F-1	F-2	F-3	F-4	F-5
LUT	0.5	0.5	0.5	0.5	0.5
EGT	0.5	0.34	0.25	0.16	-
EC	-	0.16	0.25	0.34	0.5
Acetone	10	10	10	10	10
Tween 80	0.6	0.6	0.6	0.6	0.6
Distilled Water (Up to)	100	100	100	100	100

Note: * LUT, EGT & EC Quantities are in grams; acetone, water and Tween 80 volume is in mL.

**Table 2:** Physicochemical evaluation of Luteolin loaded gastric floating microsponge.

Parameters (%)	Formulations				
	F-1	F-2	F-3	F-4	F-5
Product Yield	97.59 ± 0.54	78.01 ± 0.78	64.45 ± 0.83	40.43 ± 0.63	30.94 ± 0.33
Drug content	24.67 ± 1.76	29.5 ± 1.80	33.67 ± 1.89	28.33 ± 1.04	26 ± 0.87
Entrapment efficiency	49.33 ± 3.51	59 ± 3.6	67.33 ± 3.79	56.67 ± 2.08	52 ± 1.73
In-Vitro floating (h)	>8	>8	>8	>8	>8
Particle size (µm)	3.36	3.40	3.42	3.57	3.61

Each study was performed in triplicate and data shown as mean ± SD (*n* = 3).

**Table 3:** MICs of test compounds and their duration of action against *H. pylori* (ATCC 43504).

Test Compounds	MIC (µg/mL)	Duration of Action (at 2× MIC) Hours
Luteolin	2	24
Luteolin gastric floating microsponge (F-3)	4	48

## Data Availability

Not applicable.
